# ST-segment elevation in a patient with cardiac lymphoma

**DOI:** 10.1007/s12471-019-01345-5

**Published:** 2019-11-22

**Authors:** J. P. Guimarães, J. Trigo, F. Fonçalves, J. I. Moreira

**Affiliations:** Cardiology Department, Tras-os-Montes and Alto Douro Hospital Centre, Vila Real, Portugal

A 78-year-old man presented with a 1-month history of exertional dyspnoea, anorexia, weight loss and night sweats. On physical examination the patient had a cachectic appearance and multiple 2‑ to 4‑cm-diameter skin lesions (Fig. 1a). Electrocardiography (Fig. 1b) showed ST-segment elevation in leads V_1_ and V_2_ and ST-segment depression from V_3_ to V_6_ with no dynamic changes in serial electrocardiograms. Transthoracic echocardiography revealed large heterogeneous masses adhered to the myocardium with no clear cleavage plane, an intermediate echogenicity and involving the right ventricle, the atrioventricular groove and the large vessels (Fig. 1c; Electronic Supplementary Material, videos 1–3). He was admitted and a skin biopsy was performed which was compatible with diffuse large B‑cell lymphoma.

**Fig. 1:**
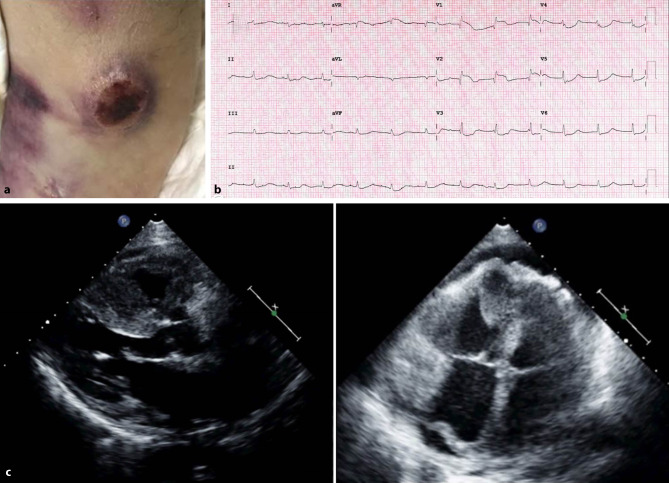
**a** Skin lesion with a necrotic centre. **b** Electrocardiogram. **c** Echocardiogram showing masses involving the right ventricle, the atrioventricular grooves and the large vessels. The masses were more exuberant in the right ventricle and right ventricular outflow tract with a maximum width of 25 mm near the right ventricular apex

Cardiac lymphoma usually occurs in the context of metastatic involvement and can manifest in a variety of ways, depending on the location of the masses [1, 2]. This patient had no acute myocardial infarction criteria [3]. We hypothesise that the ST changes were explained by the infiltration or displacement of the myocardium by the tumour, mimicking underlying myocardial infarction.

## Caption Electronic Supplementary Material

Echocardiographic 2D cine loops (parasternal long axis, parasternal short axis and four-chamber views)

Echocardiographic 2D cine loops (parasternal long axis, parasternal short axis and four-chamber views)

Echocardiographic 2D cine loops (parasternal long axis, parasternal short axis and four-chamber views)
